# Genetic and Epigenetic Intra-tumour Heterogeneity in Colorectal Cancer

**DOI:** 10.1007/s00268-016-3860-z

**Published:** 2017-01-17

**Authors:** Huw Geraint Jones, Gareth Jenkins, Namor Williams, Paul Griffiths, Phil Chambers, John Beynon, Dean Harris

**Affiliations:** 1 Department of Colorectal Surgery, Singleton Hospital, Abertawe Bro Morgannwg University Local Health Board, Sketty Lane, Swansea, SA2 8QA UK; 2 Department of Pathology, Singleton Hospital, Abertawe Bro Morgannwg University Local Health Board, Sketty Lane, Swansea, SA2 8QA UK; 3 Institute of Life Science, Singleton Park, Swansea University, Swansea, SA2 8PP UK; 4 Leeds Institute of Cancer and Pathology, Wellcome Trust Brenner Building, Level 4, St. James’s University Hospital, Leeds, LS9 7TF UK; 5 16 Forrest Road, Canton, Cardiff, CF5 1HR UK

## Abstract

**Introduction:**

Colorectal cancer (CRC) is a highly heterogeneous disease, with pathologically similar cancers having completely different responses to treatment and patient survival. Intra-tumour heterogeneity (defined as distinct morphological and phenotypic differences) has recently been demonstrated to be an important factor in the development and behaviour of cancer cells and can be used to determine response to anticancer therapy.

**Method:**

Patients with resected CRC had DNA extracted from eight defined tumour areas which were analysed for two genetic mutations (BRAF and KRAS) and one epigenetic trait (CpG island methylator phenotype/CIMP). Normal adjacent tissue was studied as control.

**Results:**

Twelve patients with CRC were included. Intra-tumoural heterogeneity for KRAS mutation was seen in 2 patients (17%). There was no statistical evidence of CIMP status heterogeneity (*p* = 0.85), but 6 of the 12 patients (50%) demonstrated at least one heterogeneous area within the tumour.

**Discussion:**

Intra-tumoural heterogeneity for both genetic and epigenetic factors in CRC is more prevalent than previously thought in Stage II and Stage III CRC. This study provides new insight into epigenetic heterogeneity of CRC and supports the development of a more targeted biopsy strategy to support expansion of personalised treatment.

## Introduction

Intra-tumour heterogeneity is defined as the distinct morphological and phenotypic differences within a tumour. This includes cellular morphology, gene expression, proliferation, metabolism, motility and metastatic potential [1]. It has been known since the earliest days of cancer cell biology that phenotypic heterogeneity of cancer cells within a tumour exists; however, only very recently have high-resolution genome-wide studies confirmed a great amount of heterogeneity within individual cancers and population diversity in mutations involving quantitative trait loci [2, 3]. This diversity likely represents a Darwinian, natural selection model of the clonal evolution of cancer biology [4]. There are two models used to explain intra-tumour heterogeneity: the cancer stem cell theory and the clonal expansion theory. These are not exclusive, and both are believed to contribute to the process in varying levels across different cancer types [5].

There is no consensus around what part, or how many samples of a CRC primary tumour should be sampled in order to identify potentially prognostic molecular or histopathological characteristics of a tumour. Baisse et al. [6] performed a study of 15 patients who were treated surgically for advanced primary sporadic colorectal adenocarcinoma (Dukes’C or D) in the late 1990s. They analysed 15–20 areas within the tumour according to the degree of histological differentiation and depth of invasion of the tumour. In addition, one sample of normal colonic mucosa and lymph node metastases was taken for analysis. The sample location was recorded on a three-dimensional grid. Samples were tested for gene alterations in *KRAS, p53* and LOH in the 5q locus, and 18q locus. They found that 67% of the analysed tumours had tumour heterogeneity in at least 1 gene locus confirming significant tumour heterogeneity in advanced CRC, and recommending a different approach of tumour sampling.

The aim of this study was to establish whether a significant level of genetic or epigenetic heterogeneity could be demonstrated in patients undergoing surgery for colorectal cancer. An assessment could therefore be made on the adequacy of pre-operative biopsies in representing the whole tumour in terms of the genetic and epigenetic fingerprint, as there are currently no guidelines to support this.

## Methodology

Twelve patients having elective surgery for colorectal cancer between January and June 2014 were randomly selected in this prospective study. Two pathologists reviewed the samples and rejected them if they were too small (<5 cm), as this would have restricted the ability to sample multiple areas for analysis. None of the patients had neoadjuvant treatment (radiotherapy or chemotherapy), as this may have altered the genetic or epigenetic profile of the tumour. The decision not to give neoadjuvant therapy was made by the regional multidisciplinary team (MDT) and was not influenced by the study. This decision was based on the pre-operative staging of the tumour. There were no other exclusion criteria, and patients were randomly selected. The tumour sample demographics, site of tumour, clinical and pathological stage, type of surgery, type and duration of adjuvant therapy, as well as overall and disease-free survival were recorded. Ethical approval for this study was granted by South West Wales REC (Project Ref No.:11/WA/0256).

### Sampling strategy

**Fig. 1:**
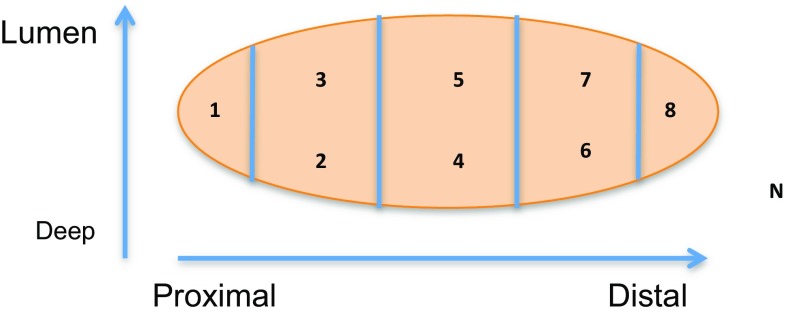
Specimen sampling template. *N normal colonic or rectal tissue adjacent to the tumour

Two pathologists from Singleton and Morriston Hospitals, Swansea, sampled the resection specimen in a two-dimensional template configuration (Fig. 1).

This resulted in eight areas of the primary tumour being sampled (proximal, proximal third deep, proximal third lumen, centre deep, centre lumen, distal third deep, distal third lumen and distal), as well as one sample of normal surrounding colonic tissue and of any metastatic lymph nodes.

### DNA extraction

Formalin-fixed paraffin-embedded (FFPE) colorectal cancer specimens were utilised for this study. Several representative 5-µm sections of the biopsy were cut and mounted unstained onto glass slides, and DNA from these tissues was obtained using the MasterPure Complete DNA and RNA purification kit (Epicentre, Illumina, Wisconsin, USA).

The quantity and quality of DNA were measured at absorbance between 230 and 320 nm using spectrophotometry (Nanodrop ND-1000 software version 3.1.2, Thermoscientific, Delaware, USA). DNA quantity was calculated by multiplying the measured concentration following spectrophotometry at 260 nm with the dilution factor. DNA was diluted to a working concentration of 20 ng/μl. Purity was further analysed by calculating the absorbance at 260 nm to absorbance at 280 nm ratio.

### Bisulphite conversion and methylation-specific PCR (MSP)

MSP was accomplished by performing bisulphite conversion of genomic DNA (Imprint DNA Modification Kit, Sigma-Aldrich, USA). The PCR products were resolved using gel electrophoresis on a 30% polyacrylamide gel. Depending on the methylation status of each CpG island, each patient could be classified as one of three epigenotypes: CIMP-High, Intermediate or Low using a two-panel approach [7, 8]. The first panel consists of SOCS1, MINT-1 and hMLH, which are associated strongly with CIMP-H. The second panel consists of NEUROG1, THBD, HAND1, ADAMTS1 and IGFBP3. CIMP status could then be determined using the following system. All samples were tested in triplicate.

- CIMP-High if ≥2/3 group 1 markers methylated
- CIMP-Intermediate if <2/3 group 1 but ≥3/5 group 2 methylated
- CIMP-Low if <2/3 group 1 and <3/5 group 2 methylated.

### KRAS and BRAF mutational analysis

Pyrosequencing analysis was performed upon the clinical specimens of this research project in collaboration with the Leeds Cancer Research UK Centre (Leeds Institute of Cancer Studies and Pathology, Clinical Sciences Building, level 6, St. James’s University Hospital, Leeds, LS9 7TF). Pyrosequencing conditions used were as previously published by this group [9]. Substitution and insertion/deletion mutations in KRAS codons 12, 13 and 61 and BRAF-600 were examined for all specimens using this method.

### Statistics

Statistical analysis was performed using SPSS version 18 (SPSS Inc, Chicago). Data were tested for normality using Kolmogorov–Smirnov test, and Student’s *t* test was used for analysis of normally distributed continuous data. Categorical variables were compared using Chi-squared [2] or Fisher’s exact test, where expected frequencies were less than 10.

### Test of CIMP heterogeneity

**Fig. 2:**
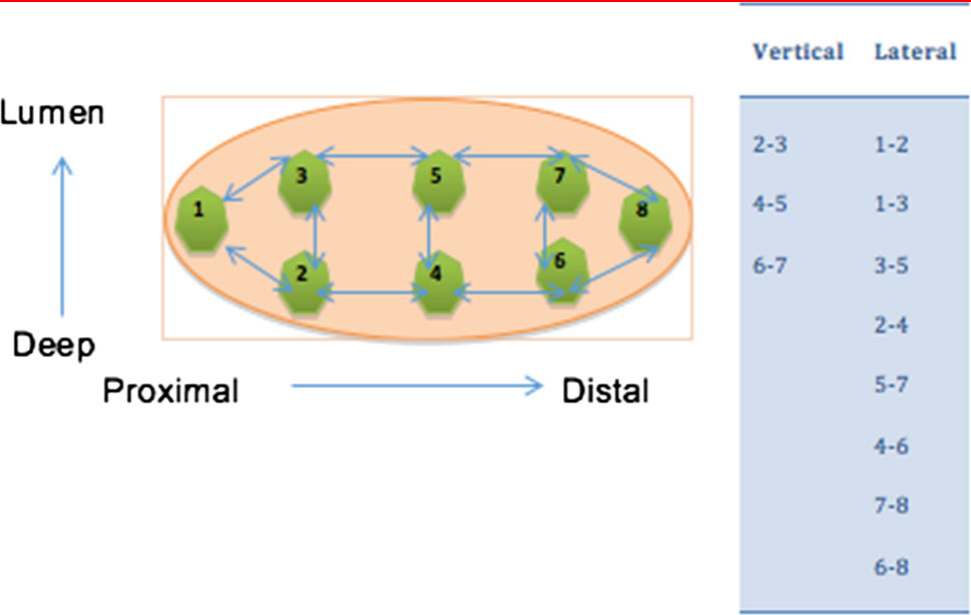
Visual representation of adjacent tissue

In the absence of a standard measurement of heterogeneity, we consulted a statistician working for the School of Medicine at Swansea University who designed the following simple metric. There are 12 tumours, each of which has been divided into eight locations (Fig. 1). The adjacency rules we imposed mean that of 28 possible pairs of locations, only 11 would be considered to be adjacent. By sampling pairs at random, we can count the number of times the methylation status matches between any two locations and build up a null distribution for the number of matches. We take as our test statistic the total number of matches in methylation status between adjacent locations in all patients and compare this to our null distribution, generated by observing randomly chosen pairs. This comparison gives us our p-value. The adjacent pairs are listed below (Fig. 2).

In total, there are 8 × 7 = 56 possible pairs from the eight observations. The data can now be used to form a histogram of “agreements”, and this will demonstrate if there is overall heterogeneity (*p* < 0.05) or homogeneity (*p* > 0.05) of the sample.

### KRAS and BRAF heterogeneity

The degree of KRAS and BRAF heterogeneity was calculated using basic probability. This probability is our *p* value since it represents the chance of seeing such agreement conditional on the null hypothesis of no association.

## Results

Twelve patients with colorectal cancer were included in the study, with 8 samples being taken from each tumour and one sample from normal surrounding tissue. Two patients (patients 4 and 7) also had lymph node metastases, which were taken for analysis. Table 1 outlines the patient and tumour characteristics for the included specimens. There were three rectal cancers, five left colon/sigmoid cancers and four right colon cancers. This included ten males and two female patients with a median age of 70 (range 56–86 years). There were 2 (17%) patients with pAJCC Stage IV disease (i.e. metastatic disease at the time of surgery), 7 (58%) patients had Stage II disease, and three (25%) patients had Stage III disease. Four (33%) patients had extramural vascular invasion (EMVI) of the tumour, and nine (66%) had moderately differentiated tumours, with one poorly differentiated and two well-differentiated tumours.

**Table 1:** Patient and tumour characteristics

Patient	Age	Sex	Tumour location	Dukes	pT	pN	M	pAJCC	EMVI	CRM +ve	Perforated	Cellular diff.
1	60	Male	Rectum	B	3	0	1	4	0	0	0	Moderate
2	67	Male	Splenic	C1	3	1	0	2	1	0	1	Well
3	80	Female	Caecum	B	3	0	0	2	1	0	0	Poor
4	65	Female	Sigmoid	C1	4	2	0	3	1	0	0	Poor
5	86	Male	Sigmoid	C1	4	0	0	2	0	0	0	Moderate
6	76	Female	Sigmoid	B	4	0	0	2	0	0	0	Moderate
7	66	Male	Hepatic	C1	4	1	0	3	1	0	0	Moderate
8	56	Male	Caecum	C1	3	0	0	2	0	0	0	Moderate
9	69	Male	Ascending	B	3	0	0	2	0	0	0	Moderate
10	79	Male	Sigmoid	C1	3	1	1	4	0	0	0	Moderate
11	75	Male	Rectum	B	3	0	0	2	0	0	0	Moderate
12	71	Male	Rectum	C1	3	1	0	3	0	0	0	Moderate

*pAJCC* pathological American Joint Committee on Cancer Grade, *EMVI* extramural vascular invasion, *CRM* circumferential resection margin, Cellular *diff* cellular differentiation, *M* male, *F* female, *R* rectum, *Sp* splenic flexure, *C* Caecum, *Si* sigmoid, *Hp* hepatic flexure, *As* ascending colon, *W* well, *Md* moderate, *P* poor

Data were also collected on the overall survival (OS) and disease-free survival (DFS) of the 12 patients with a note made of the presence of local or systemic recurrence. As this was a prospective study, the length of follow-up was relatively short (median 12.5 months). As the number of included patients is small, no analysis of survival against tumour factors was performed.

### *KRAS* and *BRAF* analysis

**Fig. 3:**
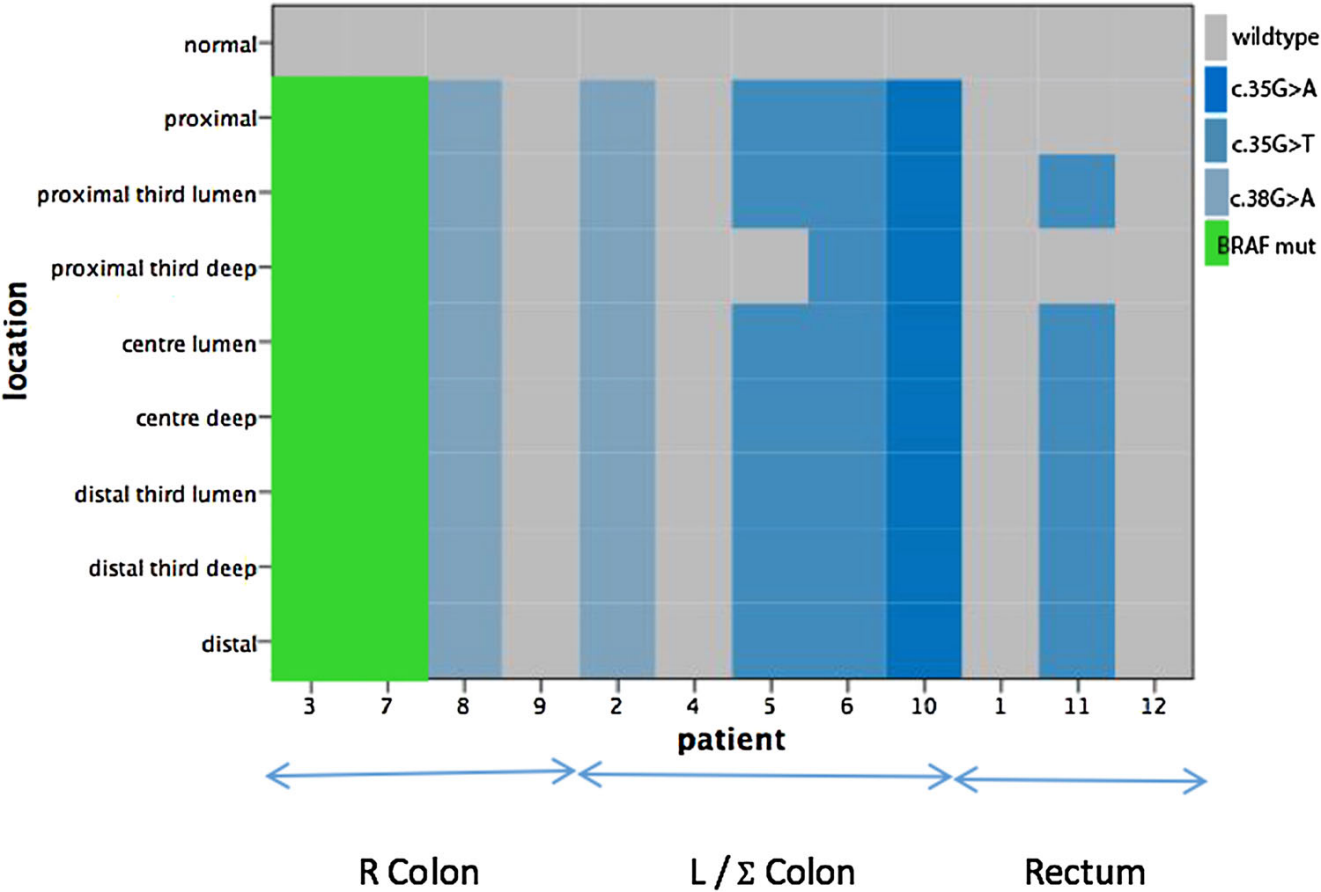
Distribution of the BRAF and KRAS mutations in the 12 patients. Patients were ranked from proximal tumour location (caecum, patient 3) to distal (rectal, patient 12)

*KRAS* and *BRAF* mutations, determined by pyrosequencing as previously described, were found to be exclusive of each other, with no patient having a mutation of both genes regardless of tumour location. Two patients had a BRAF mutation (both c.1799T > A/V600E mutations), and both had tumours located in the right colon. Five of the six *KRAS* mutant tumours were in the left colon, sigmoid colon or rectum (Fig. 3).

Ten of the twelve patients (83%) had a homogenous distribution of both *KRAS* and *BRAF* genotypes. All *BRAF* mutations (2 patients) were homogenous in nature, where two of six (33%) patients with a *KRAS* mutation having evidence of intra-tumour heterogeneity. Two patients (4 and 7) had lymph node metastases, and the primary tumour and lymph node had same genotype in both cases. For ease of interpretation, patients have been ordered by the tumour location (right colon: patients 3, 7, 8 and 9; left colon: patients 2, 4, 5, 6 and 10; rectum: patients 1, 11 and 12).

### CIMP status

**Fig. 4:**
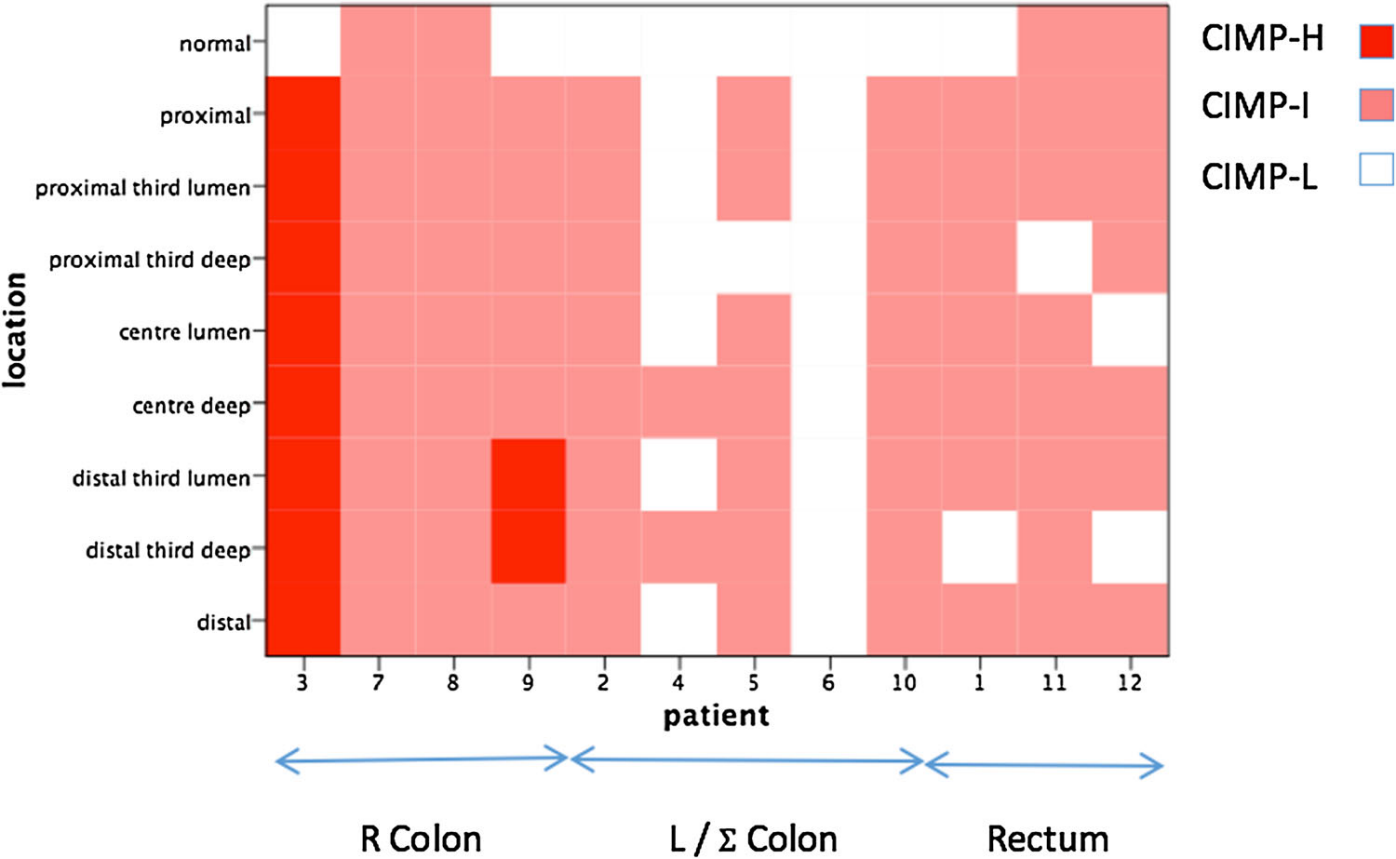
CIMP status and tumour location

Figure 4 demonstrates the CIMP status of all nine locations for all 12 patients. Both the patients with CIMP-H tumours had right colon cancers. It is also evident that the normal tissue surrounding the tumour had a lower CIMP classification than the tumour. Six patients (50%) have homogenous tumours in terms of CIMP status by this measure. There were four normal mucosa specimens (patients 7, 8, 11 and 12) that demonstrated CIMP-Intermediate status. On histopathological examination, there was no evidence of tumour cells in these specimens.

### Test of CIMP heterogeneity

**Fig. 5:**
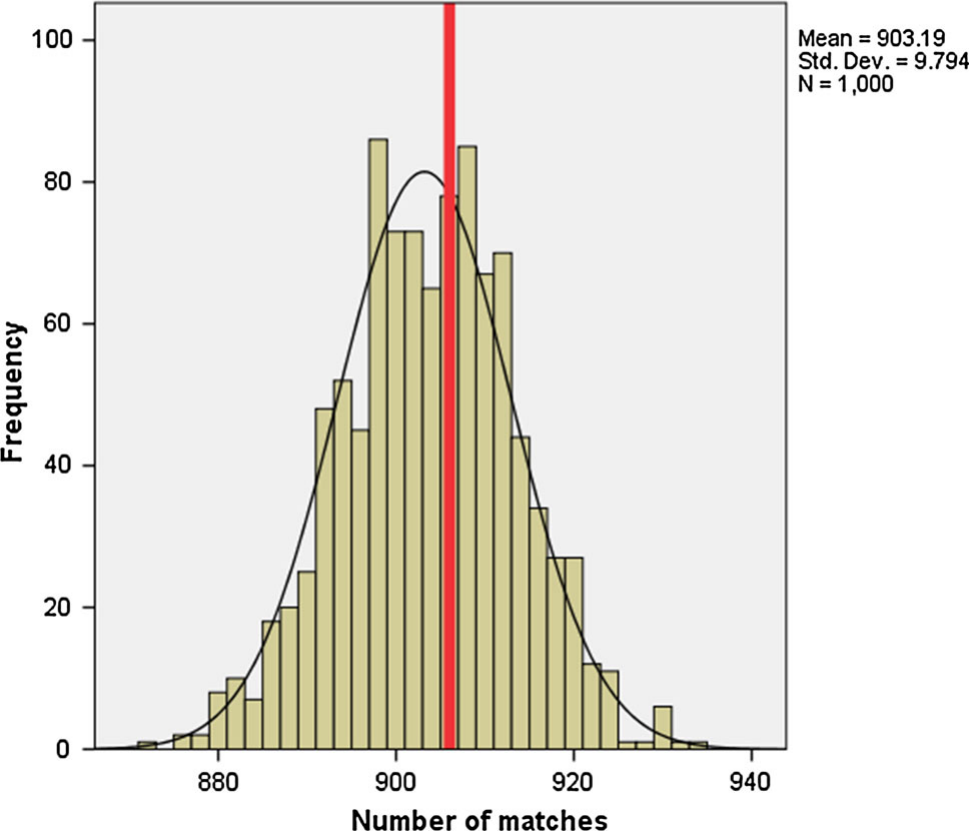
Histogram of CIMP heterogeneity

Figure 5 demonstrates that there is a relationship between adjacent pairs of tissue [*p* value = 0.85 (95% CI 0.76, 0.91)]. This suggests that the 12 tumour samples are generally homogenous in terms of CIMP classification.

### CIMP status of metastatic lymph nodes

Two patients had metastatic lymph nodes resected at the index operation (patients 4 and 7). The tumour and lymph node characteristics are shown in Table 2. There were no defining genetic or epigenetic factors in these two patients that differentiated them from patients with non-metastatic disease. Patient 4 was found to have hepatic metastases shortly after surgery, and patient 7 currently remains recurrence-free.

**Table 2:** CIMP status of metastatic lymph nodes

Patient	Normal tissue	Tumour	Lymph node metastases
4	CIMP-L	Mostly CIMP-I	CIMP-L
7	CIMP-I	CIMP-I	CIMP-I

### KRAS heterogeneity and CIMP status

**Fig. 6:**
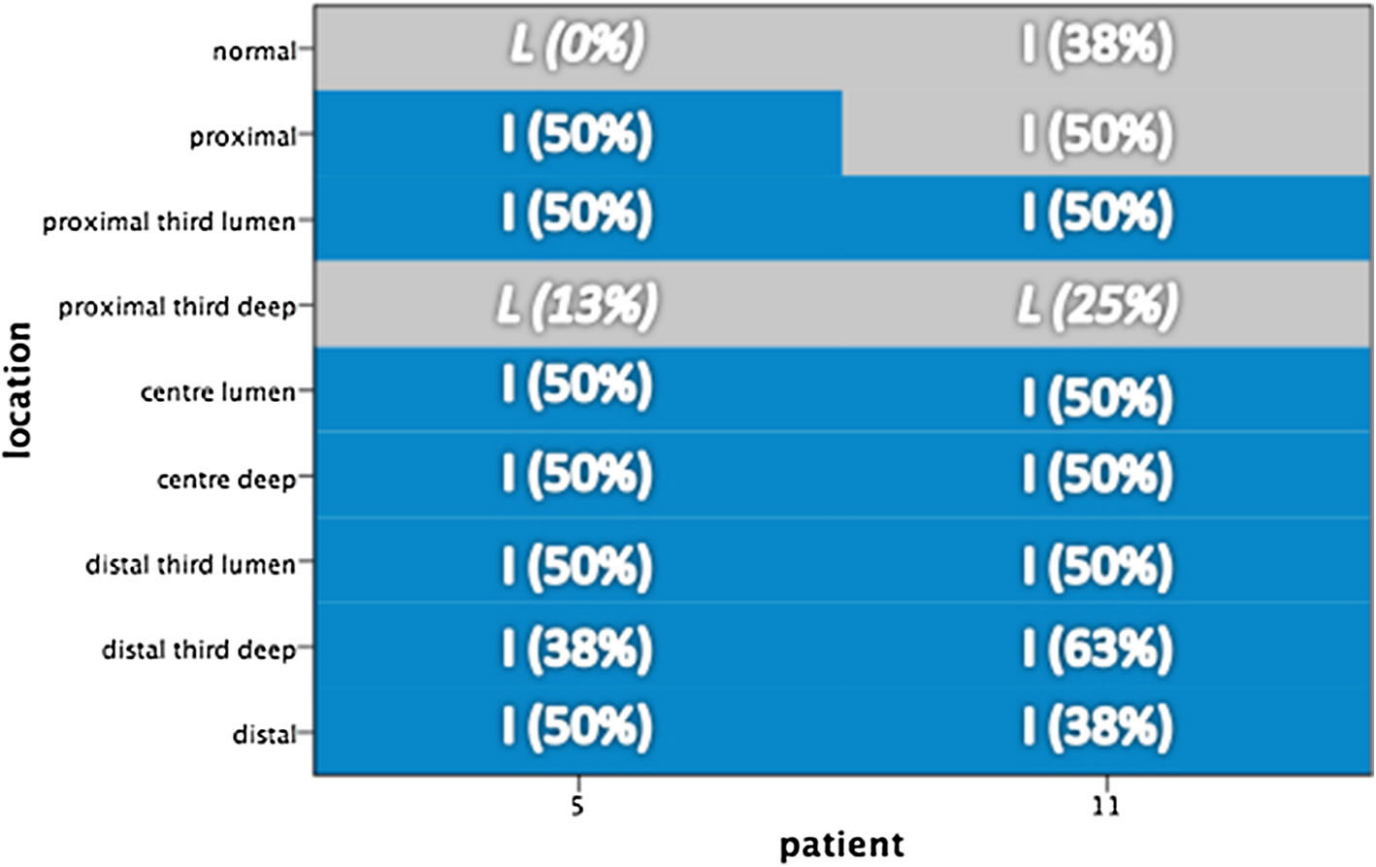
KRAS and CIMP status in patients 5 and 11. Diagrammatic representation of KRAS and CIMP status in patients 5 and 11. *Blue areas* represent KRAS mutation, and *grey areas* represent KRAS wild type. The overlaying text demonstrates CIMP status (*L* Low; *I* Intermediate) and the corresponding percentage of methylation

As previously mentioned, two patients had heterogeneous *KRAS* mutations across the tumour (patients 5 and 11). When these areas are compared to the CIMP status of those tumours, the KRAS mutation is seen in areas of hypermethylation. Figure 6 demonstrates this, where the areas within the tumour with mutant KRAS (blue areas) have higher CIMP status compared with KRAS wild type (grey areas). Given that we observed three samples with low CIMP status, the chance they would all fall on areas with KRAS wild type (of which there were only 5) can be calculated using basic probability. This probability is our *p* value since it represents the chance of seeing such agreement conditional on the null hypothesis of no association.

In patient 5, there is one area within the tumour and normal tissue proximal to the tumour that is both exclusively CIMP-L and KRAS-wt. The probability of this occurring spontaneously is 2.8% (1/9 × 1/8). In patient 11, the chance that the CIMP-L area will be in a KRAS-wt area is 33% (3/9). Given the situation, we have observed in both patients and assuming the link was random, we can assign a p-value of only around 1% (*p* = 0.009) to this event. There is therefore a significant association between the two variables, KRAS mutation and CIMP status.

If only one area of the tumour was tested for a KRAS mutation from each patient, there would be a 3.13% chance that the mutation would be missed, based on our 12-patient sample and the sample being divided into eight equal parts. If this was increased to two areas, the error rate is reduced to 0.7%, and this is reduced even further to 0.1% if 3 samples were tested.

## Discussion

It is widely recognised that there are founder genetic mutations common to all cells within a tumour from the clonal expansion theory of carcinogenesis [10, 11]. With the recent advances in massively parallel genomic sequencing, which can define the proportion of a tumour with any given mutation, there is now definite evidence of intra-tumour clonal heterogeneity [12]. The driving forces behind this are as yet unknown; however, genomic instability is thought of as a potential mechanism [13]. Much less is known about the role of epigenetics in the carcinogenesis sequence, but in the epigenetic progenitor model, DNA hypermethylation is the root cause of the genomic instability that drives the whole mechanism forward [14]. This suggests a central role of the interplay between genetic and epigenetic factors [15].

Whatever the underlying cause and exact sequence of colorectal carcinogenesis, it is becoming increasingly apparent that there are important clinical implications to the presence of intra-tumour heterogeneity. This includes a possible explanation for therapy resistance [16], a need for greater vigilance while using biopsies to diagnose the disease, and utilising the presence of heterogeneity as a biomarker [17]. An understanding of the tumour microenvironment is important for the clinician to appreciate, as it may affect our clinical practice in terms of pre-operative tumour sampling, as well as guide decisions regarding future applications of neoadjuvant and adjuvant therapies.

Richman et al. [18] studied 69 primary CRC in 68 patients and demonstrated that 10.1% patients displayed intra-tumour heterogeneity in *KRAS* codons 12, 13 and *2* or *BRAF* codon 600. Therefore, testing DNA from a single block will wrongly assign wild-type status to around 10%. These figures have been replicated by Farber et al. [19]. These papers did not explore the heterogeneity between specific locations in the tumour, as [6] had done as the samples had not been mapped at the time of formalin fixation and paraffin embedding (FFPE). Molinari et al. [20] analysed EGFR gene status and protein expression, as well as *KRAS/BRAF* mutations in 38 metastatic CRC. They found *EGFR* gene deregulation in 25 out of 36 primary tumours and 29 out of 36 metastases, *KRAS* mutations in 16 out of 37 cancers and in 15 out of 37 metastases, and *BRAF* mutations in 2 out of 36 cancers and 2 out of 36 metastases. By doing this analysis, they demonstrated that primary colorectal cancer and paired metastasis might exhibit difference with respect to EGFR pathway deregulation mechanisms, which may lead to differing response to treatment.

More recently, Fadhil et al. [21] highlighted that important decisions regarding neoadjuvant treatment are made from a small biopsy sample from the surface of the tumour. This could lead to inappropriate and costly errors in treatment choice. They demonstrated, by comparing molecular markers such as *KRAS, BRAF, PIK3CA, TP53* and MSI, that there was not a significant difference in the markers found in the biopsy sample and the resection sample. This study was on a relatively small patient sample (*n* = 20), and unfortunately, they failed to adequately explain which part of the resection sample was tested. This is a drawback of this study, as it would be expected that a homogenous result was seen if the same area of the tumour was sampled in the biopsy and resection specimens.

The results from this work suggest that the majority of colorectal cancers are largely homogenous in terms of KRAS, BRAF and CIMP status. Currently, only pre-operative biopsies for KRAS status have any bearing on treatment, as the decision to use EGFR-inhibitors is based on this result. In our sample, 2 of 6 patients with a KRAS mutation (33%) had some degree of heterogeneity found in the sample; therefore, a recommendation can be made that that at least two biopsies from different parts of the tumour be taken to take the heterogeneity into consideration.

### Relationship between genetic and epigenetic changes

Previous work by Jass [22] attempted to combine patterns of genetic and epigenetic characteristics in colorectal cancer. Although the final Jass classification is divided into five groups, he suggested that that CIMP-High CRC was generally associated with BRAF mutations, KRAS wild type, a right colon tumour, female predominance and MSI-H. In contrast, low levels of DNA hypermethylation (CIMP-Low) were associated with left-sided cancers, a male predominance, KRAS mutation, BRAF wild type and MSS. This study generally found this to be true, with both CIMP-H tumours being located in the right colon and having BRAF mutations and KRAS wild type. The presence of CIMP-L was only seen in a heterogeneous fashion in the left colon and rectum, and this was associated with *KRAS* mutation and *BRAF* wild type. It was not possible to formally classify the patients into a definite Jass Grouping [22], as no data were available on the MSI status.

From the relationship seen between the genetic and epigenetic factors, we can postulate that the epigenetic changes have occurred later on than the KRAS mutation, as there was a strong association between the unmethylated areas with KRAS-wt. This phenomenon was only seen in two patients, and larger numbers would be needed to expand on this theory.

### Limitations of this study

The large amount of methylation-specific PCR needed (a minimum of 9 samples per patient, with 8 different gene promoter regions to test on each sample) limited the amount of patients in this study to 12. It is possible that we did not have a representative sample of the general population because of this. Other limitations included the necessity of selecting patients with advanced local disease (pT3/4) that were not selected for neoadjuvant therapy. The guidelines state that there is no evidence to support pre-operative chemotherapy versus surgery alone in patients with locally advanced colon cancer; however, in the patients with rectal cancer, chemoradiotherapy before surgery is recommended for patients with high-risk locally advanced rectal cancer to allow tumour response and shrinkage [23]. Of the three patients with rectal cancer included in the current study, none had any high risks outlined in the NICE guidelines, as the specimen had clear resection margins, and the tumours were not encroaching on the inter-sphincteric space or the levator ani muscle.

The group of CIMP panel markers that were selected also limited the results, and it is possible that this is not a true representation of the CIMP status of the whole genome. Recent work has established that single-cell genome-wide bisulphite sequencing is a valid method of measuring DNA methylation in mice [24]. This technique could be utilised in humans to aid in the difficult task of measuring epigenetic heterogeneity in cancer. Analysis of further mutations (e.g. *TP53 and PIC3CA)* and microsatellite instability may have provided further insight into the cause of intra-tumour heterogeneity.

## Conclusion

This pilot study involving 12 patients exploring intra-tumour heterogeneity of colorectal cancer has demonstrated relative homogeneity of genetic factors (BRAF and KRAS mutations), as well as epigenetic homogeneity (in terms of DNA methylation). It has analysed data from a relatively small number of patients, but importantly, the location of the biopsies in the tumour was decided according to a defined template, which investigated mutations based on tumour topography. It has also revealed interesting patterns of association of CIMP and KRAS/BRAF mutation in keeping with the work of Jass [22]. This work further aids our understanding of the complex process of tumourigenesis, and the interweaving role of the genetic and epigenetic factors.

Clinicians should be aware that at least two pre-operative biopsies be taken to avoid mis-sampling when assessing KRAS status. This number may well increase in future if the CIMP status is found to be clinically significant as a prognostic marker.

Current research is focusing on both KRAS and BRAF as predictive and diagnostic biomarkers in patients with metastatic disease treated with anti-EGFR therapies, such as panitumumab and cetuximab [25–27]. As these therapies become more sophisticated, and the era of personalised medicine arrives, it becomes increasingly important that we correctly assess the genetic and epigenetic profile of the disease. Further work in this area should focus on testing a greater number of samples, as well as to explore the heterogeneity in both early and late disease.
